# Multiple Regulatory Networks Are Activated during Cold Stress in *Medicago sativa* L.

**DOI:** 10.3390/ijms19103169

**Published:** 2018-10-15

**Authors:** Qiang Zhou, Dong Luo, Xutian Chai, Yuguo Wu, Yanrong Wang, Zhibiao Nan, Qingchuan Yang, Wenxian Liu, Zhipeng Liu

**Affiliations:** 1 The State Key Laboratory of Grassland Agro-ecosystems, Key Laboratory of Grassland Livestock Industry Innovation, Ministry of Agriculture, College of Pastoral Agriculture Science and Technology, Lanzhou University, Lanzhou 730020, China; zhouq2013@lzu.edu.cn (Q.Z.); luod13@lzu.edu.cn (D.L.); chaixt15@lzu.edu.cn (X.C.); wuyg16@lzu.edu.cn (Yu.W.); yrwang@lzu.edu.cn (Ya.W.); zhibiao@lzu.edu.cn (Z.N.); 2 Institute of Animal Sciences, Chinese Academy of Agricultural Sciences, Beijing 100000, China; qchyang66@163.com

**Keywords:** *Medicago sativa* L., cold stress, RNA-Seq, differentially expressed genes, antioxidant defense

## Abstract

Cultivated alfalfa (*Medicago sativa* L.) is one of the most important perennial legume forages in the world, and it has considerable potential as a valuable forage crop for livestock. However, the molecular mechanisms underlying alfalfa responses to cold stress are largely unknown. In this study, the transcriptome changes in alfalfa under cold stress at 4 °C for 2, 6, 24, and 48 h (three replicates for each time point) were analyzed using the high-throughput sequencing platform, BGISEQ-500, resulting in the identification of 50,809 annotated unigenes and 5283 differentially expressed genes (DEGs). Metabolic pathway enrichment analysis demonstrated that the DEGs were involved in carbohydrate metabolism, photosynthesis, plant hormone signal transduction, and the biosynthesis of amino acids. Moreover, the physiological changes of glutathione and proline content, catalase, and peroxidase activity were in accordance with dynamic transcript profiles of the relevant genes. Additionally, some transcription factors might play important roles in the alfalfa response to cold stress, as determined by the expression pattern of the related genes during 48 h of cold stress treatment. These findings provide valuable information for identifying and characterizing important components in the cold signaling network in alfalfa and enhancing the understanding of the molecular mechanisms underlying alfalfa responses to cold stress.

## 1. Introduction

Cultivated alfalfa (*Medicago sativa* L.) is one of the most important legume forages in the world and is the fourth most widely grown crop in the United States [1,2,3]. Known as the “Queen of the Forages”, alfalfa provides highly nutritious forage in terms of protein, fiber, vitamins, and minerals for ruminant animals [4]. Thus, it has considerable potential as a valuable forage crop for livestock. In addition, it also has potential as a sustainable, cellulosic feedstock for ethanol production [4,5]. Additionally, alfalfa can improve soil quality, promote wildlife diversity, and provide its own nitrogen fertilizer through symbiotic nitrogen fixation. Therefore, it can be used for ecological restoration and can provide environmental benefits [4,5]. Alfalfa plantation areas in China are distributed in 14 provinces throughout the northern region of the country, and freezing temperatures are a major factor affecting alfalfa growth and limiting its productivity and survival throughout this range, particularly in the high altitude and northern regions of China [6]. Moreover, significant economic losses result from unusual sudden temperature changes in winter and later cold spring events. Fall dormancy (FD) is defined as an adaptive characteristic related to biomass production and winter survival in alfalfa, and it occurs with a decreasing temperature and day length [7]. For practical purposes, the dormancy level of alfalfa cultivars is determined according to a standard test, and it is classified into three groups: Dormant (FD 1–3), semi-fall dormant (FD 4–6), and non-dormant (FD > 6). Briefly, dormant cultivars produce short and prostrate shoots in autumn and possess high winter hardiness, whereas less dormant cultivars produce a higher yield in fall but risk winterkilling [7,8]. Therefore, understanding the molecular mechanism of cold stress signal transduction pathways is important for breeders to improve the cold tolerance and fall dormancy of alfalfa.

Plants undergo various changes in response to cold stress. A key response to cold is growth repression, which may be utilized to reallocate resources from growth to processes that increase cold stress resistance [9]. Cold stress also results in plant surface lesions, discoloration, tissue breakdown, accelerated senescence, and faster decay [10,11]. In addition to the changes of morphology, dramatic biochemical and physiological changes were observed. These include changes in the characteristics of the plasma membrane, the synthesis of cryoprotectant molecules, such as soluble sugars, glycine, and proline, and an increase in the scavenging activity of reactive oxygen species [12,13,14], which are all dedicated to reducing the damage caused by cold temperatures or cellular freezing.

Plant adaptation to cold or other stress conditions relies on the activation of cascades of molecular networks involved in signal transduction and the expression of specific stress-related genes and metabolites [15]. The most extensively researched freezing tolerance pathway involves a class of the C-repeat binding factor (CBF) and dehydration responsive element-binding (DREB), which specifically bind to cis-elements in the promoters of *COLD-RESPONSIVE* (COR) genes and activate their expression [16]. The CBF signaling pathway can increase freezing tolerance, which was demonstrated to exist in numerous plants, such as thale cress (*Arabidopsis thaliana*) [17], cauliflower (*Brassica oleracea* var. *botrytis*) [18], and peach (*Prunus persica*) [19]. A recent report suggested that CBFs may play an important role in the regulation of freezing tolerance in alfalfa, based on the expression of C-repeat binding factor-like (CBFl) genes [20]. In addition, it is clear that hormones act as central regulators of cold stress responses in plants. For example, abscisic acid (ABA) is an isoprenoid hormone that plays an important role in seed dormancy, abscission, and abiotic stress signaling [21]. Moreover, the exogenous application of ABA enhances cold tolerance, and ABA mutants show a weaker cold resistance. However, it was previously hypothesized that ABA does not act on CBF expression. Thus, it was proposed that ABA-controlled cold responses are regulated by CBF-independent means [22]. However, recent research suggested that ABA may affect the expression of COR genes, by controlling CBF transcription, and may play a more central role in the cold stress tolerance of plants [23,24].

Recently, next-generation sequencing (NGS) has dramatically improved the efficiency of transcriptome data collection, which has clear advantages over microarray methods and has been used in gene discovery and regulatory network studies. Moreover, it is particularly useful for species that have not yet been sequenced. Based on RNA-Seq platforms, genome-scale transcriptome analyses were used to identify cold-stress-responsive genes in crowtoe (*Lotus japonicus*) [25], cassava (*Manihot esculenta*) [26], radish (*Raphanus sativus*) [27], patience dock (*Rumex patientia*) [28], peach [29], and Chinese yew (*Taxus chinensis*) [30]. Moreover, a recent study reported that two standard varieties of alfalfa (dormant and non-dormant) were sequenced using synthesis (SBS) technology, and several differentially expressed genes that were potentially related to fall dormancy were identified [31]. Other researchers performed the first assessment of the transcript level using high-throughput transcriptome sequencing and digital gene expression analysis of the crown buds of different fall dormancy grades from alfalfa, which provides a valuable resource for future studies on functional genomics of the freezing stress in plants [6]. However, the information regarding the genetic response to cold stress is limited, because only crown buds from alfalfa were used for RNA sequencing in this study. Therefore, the entire seedling of alfalfa under cold conditions (4 °C) was collected for RNA sequencing in our study to obtain additional genetic information about the response to cold stress. The results obtained in the present study will extend knowledge of the genetic basis of the alfalfa response to cold stress at the transcription level and will also provide novel gene resources for breeders to improve the cold tolerance and fall dormancy of alfalfa.

## 2. Results and discussion

### 2.1. Cell Membrane Stability, Lipid Peroxidation, and Photosynthesis

**Figure 1:**
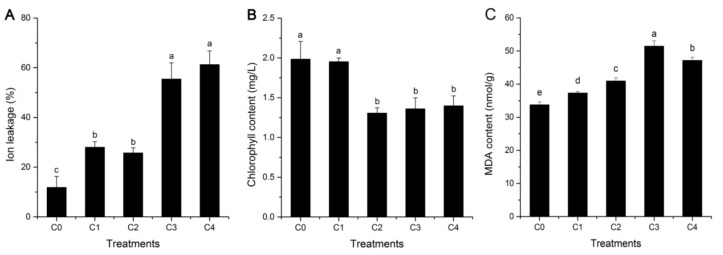
Ion leakage, chlorophyll, and malondialdehyde (MDA) content in leaf tissues of alfalfa, grown under normal and cold stress conditions. (**A**) Ion leakage, (**B**) chlorophyll content, (**C**) MDA content. Leaf tissues were sampled from plants grown under normal conditions and from plants subjected to 4 °C for 2, 6, 24, and 48 h. C0, C1, C2, C3, and C4 represent 0, 2, 6, 24, and 48 h, respectively. Each value represents the mean of three replicates ± SD (standard deviation), shown by a vertical error bar. Different letters above the bars indicate significant differences at the 0.05 level, according to Duncan’s multiple range test. In addition, a, b, c, d, e indicate the result of one-way analysis of variance, and them can reflect significant levels of difference between treatments.

The electrolyte leakage level, an important indicator of tissue and membrane integrity, was determined to investigate the cell membrane stability of alfalfa under cold stress. As shown in Figure 1A, the levels of electrolyte leakage were higher in the four groups under cold treatment, compared with those under a normal temperature, and a significant increase was observed in C3 and C4. Moreover, the chlorophyll contents of alfalfa materials were measured to reflect the change of photosynthesis between untreated and treated samples. In the present study, a decrease in chlorophyll content was observed in four groups (C1, C2, C3, and C4, which represent 2 h, 6 h, 24 h, and 48 h, respectively) under cold stress, compared with the control, but a trend appeared after C2 (Figure 1B). There were significant differences (*p* < 0.05) in the total chlorophyll contents of alfalfa leaves between three cold treatments (C2, C3, and C4) and the control (C0), whereas it was not observed in C1. These similar results have also appeared in previous reports on the physiological and biochemical responses of three grapevine cultivars with different levels of cold tolerance [32]. In addition, lipid peroxidation is induced by cold stress in many plants, and malondialdehyde (MDA) content can be used to evaluate the extent of lipid peroxidation, which further reflects the extent of oxidation injury and cellular damage. The levels of MDA content were higher in the four cold treatments, compared with the control (C0), and showed significant differences (*p* < 0.05) (Figure 1C), which is consistent with the results of a previous report on bermudagrass (*Cynodon dactylon*) under cold stress [33]. Therefore, these results suggested that ion leakage, total chlorophyll, and MDA content are some reliable signs that injury has occurred in an alfalfa seedling.

### 2.2. De Novo Assembly of Transcriptome and the Functional Annotation of Unigenes

RNA samples from the intact seedling, for the four treatments at different time points (C1, C2, C3, and C4) and one control (C0, without cold stress), were used to construct cDNA libraries. A total of 318,619,289 raw reads were ultimately obtained from the 15 libraries (three libraries for each treatment) (Table S1). A total of 305,471,639 clean reads remained after removing the adaptor sequences, ambiguous nucleotides, and low-quality sequences. Finally, 15.3 GBase of data were obtained, and each library was longer than 1 G. A total of 50,809 unigenes were assembled, with an average length of 2541 bp, and 45,696, 47,059, 45,758, 45,779, and 46,691 unigenes were identified for the C0, C1, C2, C3, and C4 groups, respectively (Table S1). The average length of the unigenes was longer than the length of the transcriptome sequences obtained from other plants, such as wheatgrasses (*Campeiostachys nutans*, 635 bp) [34], common vetch (*Vicia sativa*, 772 bp) [35], Siberian wildrye (*Elymus sibiricus*, 645 bp) [36], and Chinese yew (*Taxus chinensis*, 598 bp) [30]. Moreover, the length of these 50,809 unigenes ranged from 312 to 8445 bp, with an N50 and N70 length of 2244 and 3015 bp, respectively (Figure S1). These average and N50 lengths in our study were longer than those previously reported in alfalfa crown bud transcriptome analyses [6]. These results may be a consequence of using data from the previous transcriptome sequencing, by PacBio ISO Sequencing in our laboratory, as a reference genome for sequence assembly in this study. The relationship between the unigenes in the four treatments and the control is shown in Figure S2. In addition, the reads of this study have been deposited in the NCBI SRA database (SRR7091780~94, one number for each library).

For the functional annotation of all unigenes, BLASTx searches (*E*-value ≤ 10^−5^) were performed to search for the final unigene set against three public databases, including the NCBI non-redundant protein sequences (Nr), Gene Ontology (GO), and Kyoto Encyclopedia of Genes and Genomes (KEGG). Of these 50,809 unigenes, 21,947 (43.20%), 45,138 (88.84%), and 11,476 (22.59%) unigenes were successfully annotated in the Nr, GO, and KEGG databases (Table S2), respectively. A total of 1784 (3.51%) unigenes were annotated in all three databases, and 50,123 (98.65%) unigenes were annotated in at least one database, which was higher than that identified in previous alfalfa sequencing reports [6,37,38]. These unannotated unigenes may represent untranslated regions, non-coding RNAs, mis-assembly, or alfalfa-specific genes [35,37]. Additional details about the annotation of the four treatments and one control are provided in Table S2. In addition, GO assignments were used to classify the functions of the predicated alfalfa genes, expressed in response to cold stress. In this study, 50,809 unigenes were assigned to a total of 8332 annotations and 44 GO terms according to the sequence homology, which was grouped into three main categories (Figure S3).

### 2.3. Quantitative Real-Time PCR Verification

**Figure 2:**
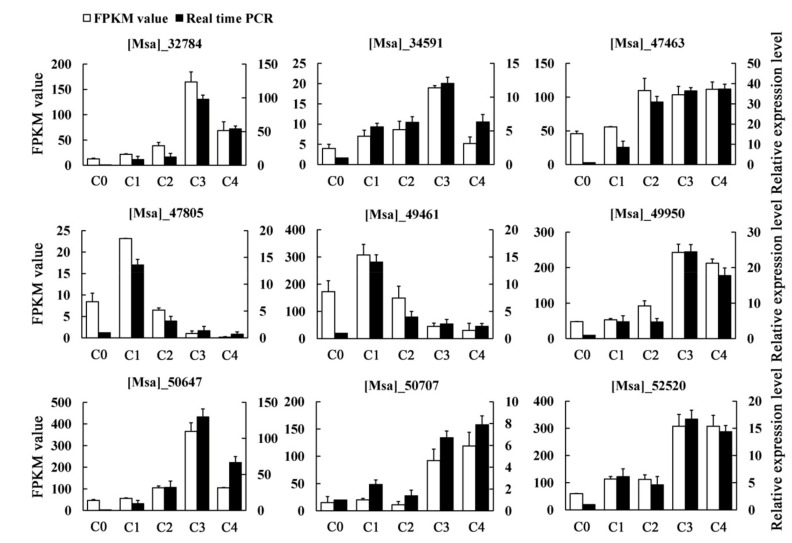
The expression patterns of nine randomly selected genes, identified by RNA-Seq, were validated by qRT-PCR. White bars indicate the transcript abundance change based on the Fragments Per Kilobase per Million Fragments mapped (FPKM) values of the RNA-Seq analysis (left y-axis). Black bars represent the relative expression levels determined by qRT-PCR (right y-axis). Error bars indicate standard errors of the means (*n* = 3).

To verify the reliability and reproducibility of the RNA-seq analysis, nine unigenes were randomly selected for quantitative real-time PCR (qRT-PCR) validation (Table S3). In our analysis, the expression of these selected unigenes in our transcriptome data were generally consistent with the qRT-PCR results, indicating that our RNA-Seq data were reliable (Figure 2).

### 2.4. Identification and Clustering Analysis of Differentially Expressed Genes (DEGs)

**Figure 3:**
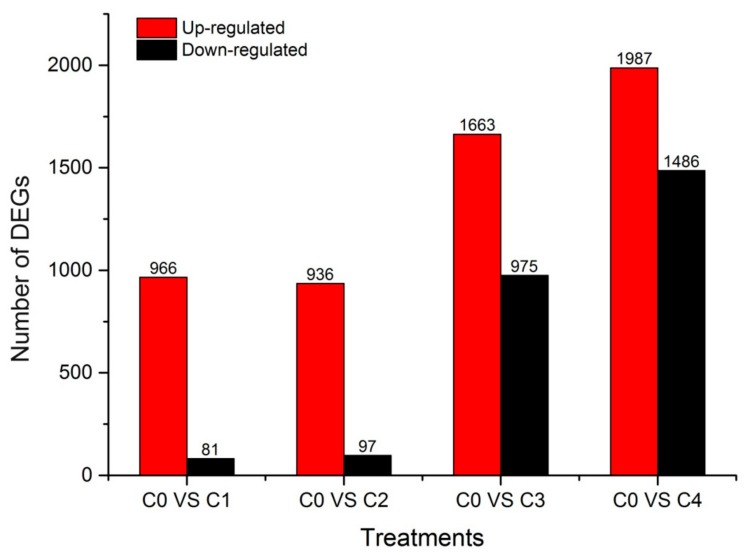
Summary of the differentially expressed unigenes (DEGs). C0, C1, C2, C3, and C4 represent 0, 2, 6, 24, and 48 h, respectively. The red column indicates the up-regulated gene, and the black column indicates the down-regulated gene.

**Figure 4:**
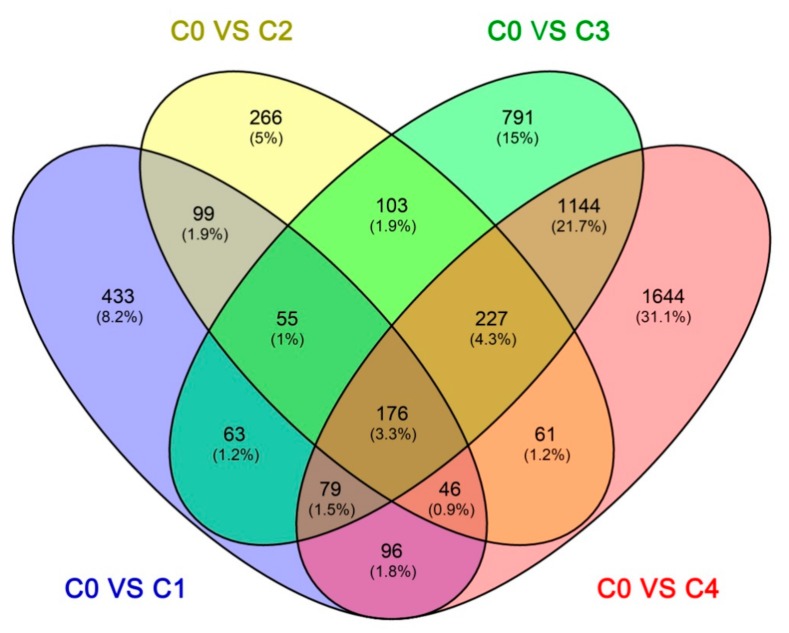
The number of DEGs expressed at one cold stress time point and at overlapping time points, compared with the control. C0, C1, C2, C3, and C4 represent 0, 2, 6, 24, and 48 h, respectively.

The Fragments Per Kilobase per Million Fragments mapped (FPKM) values from the different cold treatment time-point libraries were collected and analyzed to investigate the changes in gene expression and understand the critical genes involved in the alfalfa response to cold stress. Differential expression analyses revealed that 5283 unigenes were expressed differently, between the control and cold-stressed samples, at a fold-change (FC) ratio of more than 4 (|log2FC| > 2) (Table S4). The number of differentially expressed genes (DEGs) in our study was lower than that previously reported in alfalfa crown bud transcriptome analyses, which obtained 5605 DEGs using the ordinary standard of absolute log2-fold change ≥ 1 [6]. Of these 5283 unigenes, 1147 (966 up-regulated and 81 down-regulated), 1033 (936 up-regulated and 97 down-regulated), 2638 (1663 up-regulated and 975 down-regulated), and 3473 (1987 up-regulated and 1486 down-regulated) DEGs responded to C0 stress within C1, C2, C3, and C4, respectively (Figure 3). The number of DEGs in C3 and C4 were more than C1 and C2, and the number of down-regulated DEGs was less than the number of up-regulated DEGs in every library, particularly in C1 and C2. This is consistent with a result from previous reports in other plants under cold stress, such as rice (*Oryza longistaminata*) [39], patience dock [28], and crowtoe [25]. Furthermore, there were 433, 266, 791, and 1644 DEGs specifically modulated in C1, C2, C3, and C4, respectively. Interestingly, 176 DEGs were common to all four time points, suggesting that these genes were continuously significantly modulated during the 48-h cold stress treatment (Figure 4).

**Figure 5:**
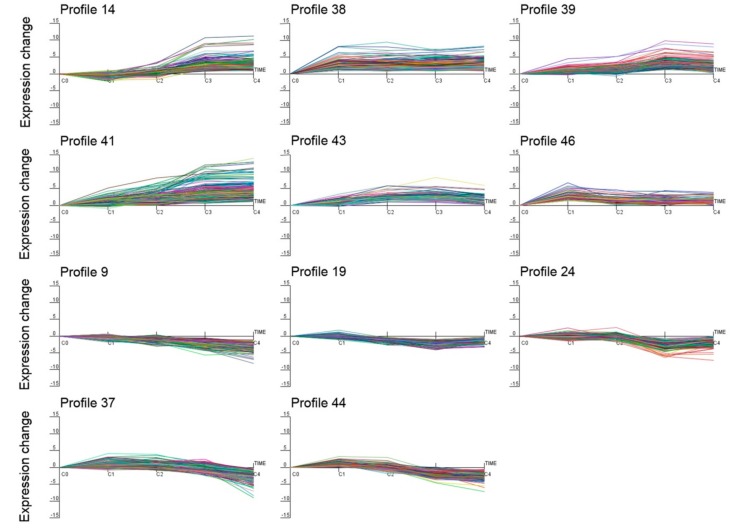
Differentially expressed gene expression profiles. The x-axis represents different cold processing points, and the y-axis represents the expression change. Profiles 14, 38, 39, 41, 43, and 46 are up-regulated patterns, and profiles 9, 19, 24, 37, and 44 are down-regulated patterns.

Using Short Time-series Expression Miner (STEM) software, the expression data υ were normalized to 0, log_2_ (υC1/υC0), log_2_ (υC2/υC0), log_2_ (υC3/υC0), and log_2_ (υC4/υC0) to examine the expression profiles of the 5283 DEGs. As a result, all 5283 DEGs were clustered into 11 profiles (*p* value ≤ 0.05), including six up-regulated patterns (profiles 14, 38, 39, 41, 43, and 46) and five down-regulated patterns (profiles 9, 19, 24, 37, and 44) (Figure 5). In addition, the hierarchical clustering of all DEGs, with expression differences in the four treatments and control, was performed using Cluster 3.0 software (Figure S4).

### 2.5. GO Functional Analysis of the DEGs

A total of 35 GO categories were assigned to the 5283 DEGs that responded to cold treatment (Figure S5). ‘‘Cell’’ and ‘‘cell part’’ genes (675 for both, 12.78%) were the dominant categories in the cellular component category, followed by ‘‘organelle’’ (216, 4.09%), “macromolecular complex” (93, 1.76%), and ‘‘organelle part’’ genes (92, 1.74%). Regarding the biological process category, ‘‘metabolic process’’ (1369, 25.91%) and ‘‘cellular process’’ (1082, 20.48%) were the most dominant groups, followed by ‘‘biological regulation’’ (249, 4.71%), “localization” and “establishment of localization” (211 for both, 3.99%), “pigmentation” (200, 3.79%), and “response to stimulus” (157, 2.97%). In the molecular function category, a total of 1261 (23.87%) and 1132 (21.43%) unigenes were assigned to ‘‘catalytic activity’’ and ‘‘binding’’, respectively, followed by “transporter activity” (167, 3.16%). These similar results were reported in previous studies on the transcriptome sequencing analysis of alfalfa in response to freezing stress [40].

**Figure 6:**
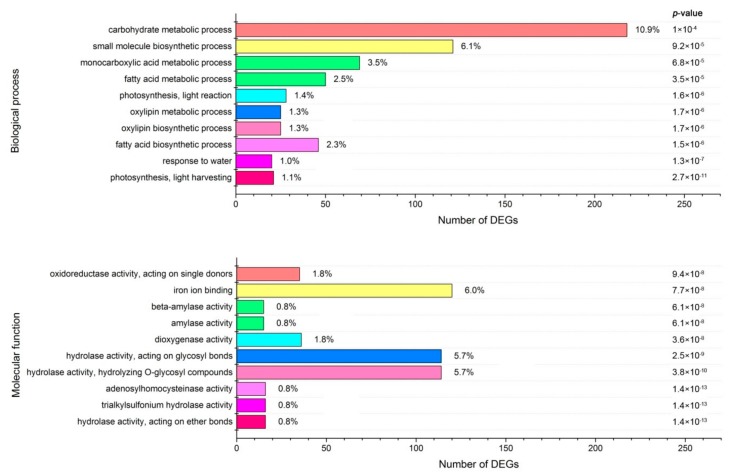
Gene ontology (GO) enrichment analysis of DEGs. The genes were assigned to two main categories: Biological process and molecular function. The names of the GO categories are listed along the y-axis. The number on the right of the column indicates the proportion of the gene number corresponding to each category in the total number of DEGs. The degree of GO enrichment is represented by the false discovery rate (FDR) value and the number of unigenes enriched in each category. The FDR value indicates the corrected p-value, ranging from 0 to 1, and an FDR value closer to 0 indicates a greater enrichment.

To identify the significantly enriched GO terms among the DEGs, we used 8332 GO terms, annotated from all unigenes as references, and performed a GO enrichment analysis of the functional significance using the agriGO website, with a *p* score cut-off of 0.05. As a result, a total of 38 GO terms were considered significantly enriched among the DEGs, and 36 and 12 GO terms belonged to ‘‘molecular function’’ (F) and ‘‘biological process’’ (P), respectively. However, no GO terms were significantly enriched in ‘‘cellular component’’ (C). The ten most significantly over-represented GO terms in each category are shown in Figure 6.

### 2.6. KEGG Pathway Enrichment Analysis of the DEGs

**Figure 7:**
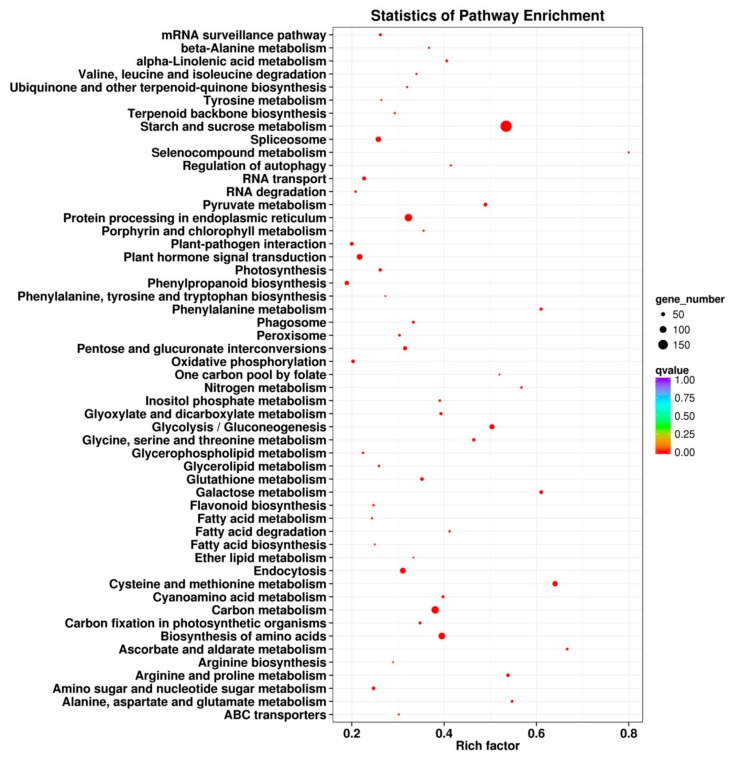
Kyoto Encyclopedia of Genes and Genomes (KEGG) pathway enrichment scatter diagram of DEGs. Only the most highly represented pathways are displayed in the diagram. The degree of KEGG pathway enrichment is represented by an enrichment factor, the FDR value, and the number of unigenes enriched in a KEGG pathway. The enrichment factor indicates the ratio of differentially expressed unigenes enriched in this pathway to the total number of annotated unigenes in this pathway. The names of the KEGG pathways are listed along the x-axis. The FDR value indicates the corrected p-value, ranging from 0 to 1, and an FDR value closer to 0 indicates a greater enrichment.

To characterize the complex biological behaviors of the transcriptome, all DEGs were subjected to a KEGG pathway enrichment analysis, using the KOBAS v2.0 website. A total of 3286 cold stress-responsive DEGs were assigned to 115 different KEGG pathways, and 53 pathways changed significantly (*q* ≤ 0.05) under cold stress treatment (Figure 7). The significantly over-represented pathways included “starch and sucrose metabolism”, “protein processing in endoplasmic reticulum”, “plant hormone signal transduction”, “cysteine and methionine metabolism”, “carbon metabolism”, and “biosynthesis of amino acids”, which is consistent with a result in a previous report on alfalfa under freezing stress [6].

### 2.7. Antioxidant Defense System-Related Genes in Cold Stress

**Figure 8:**
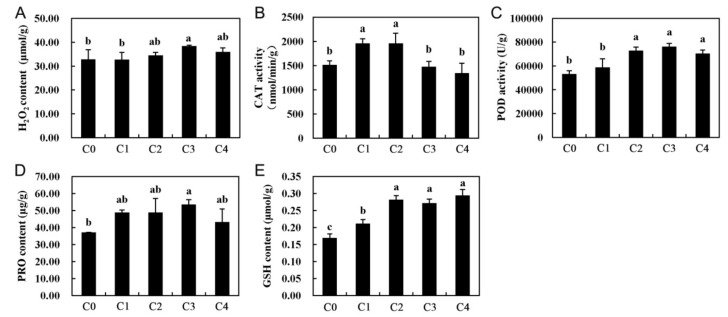
Physiological indices of whole alfalfa seedlings after 0, 2, 6, 24, and 48 h of cold stress. (**A**) H_2_O_2_ content, (**B**) CAT activity, (**C**) POD activity, (**D**) PRO content, and (**E**) GSH content. Whole seedlings were sampled from plants subjected to 4 °C for 0, 2, 6, 24, and 48 h. Additionally, C0, C1, C2, C3, and C4 represent 0, 2, 6, 24, and 48 h, respectively. Each value represents the mean of three replicates ± SD (standard deviation) shown by a vertical error bar. Different letters above the bars indicate a significant a difference at the 0.05 level, according to Duncan’s multiple range test.

Reactive oxygen species (ROS) are harmful to membranes, proteins, and biological macromolecules and accumulate in plant cells, disturbing the homeostasis of the organism [41]. ROS play important roles in regulating plant gene expression, particularly under abiotic stress conditions [42]. For example, hydrogen peroxide (H_2_O_2_) and other ROS may accumulate during cooling, which can cause oxidative stress and cellular damage to plants. In this study, H_2_O_2_ accumulated when alfalfa was exposed to cold temperature conditions (4 °C) (Figure 8A), a result consistent with those from previous reports in other plants under cold stress [43]. Catalase (CAT) and peroxidase (POD) are two types of important antioxidant enzymes that play a key role in antioxidant defense systems. CAT can degrade hydrogen peroxide into water and oxygen, and POD can also enzymatically degrade hydrogen peroxide, such that plants can resist damage from hazardous substances during metabolic processes under stress conditions, thereby showing resistance. In this study, increases in the CAT activity was observed during the first hours of cold stress (C1 and C2), but it later decreased to control levels. (Figure 8B). However, the activity of POD was further increased in all cold treated samples and showed significant differences (*p* < 0.05) (Figure 8C). Compared with the control, the activities of CAT and POD increased by varying degrees in our study (Figure 8), which is consistent with a result in previous reports on *T. chinensis* [30] and *O. longistaminata* [39]. Additionally, the DEGs related to the GO terms, “oxidoreductase activity”, “oxidation reduction”, and “dioxygenase activity”, were enriched, and the KEGG pathway enrichment analysis of the DEGs indicated that some key genes related to “peroxisome” and “oxidative phosphorylation” were also significantly enriched after cold stress treatment (Figure 7 and Figure S6), which suggested an important role for CAT and POD in response to cold stress (Figures S7 and S8), possibly to protect alfalfa from damage by ROS.

Besides CAT and POD, glutathione (GSH) is one of the most important non-enzymatic antioxidants, which can be oxidized to its oxidative form, glutathione oxidized, to decompose peroxides and can also act as a substrate of some antioxidant enzymes, such as glutathione peroxidase (GPx). Moreover, proline (Pro) can accumulate when a plant is under cold temperature conditions. It regulates cell osmotic potential, holds turgor pressure in stressed cells, and partially equips plants to undergo dehydrative stresses, with slight or no deleterious effects. In addition, the content of proline and glutathione were shown to increase under cold temperatures in previous studies [32,44]. Therefore, proline and glutathione in alfalfa, under cold stress and control conditions, were measured. As a result, both proline and glutathione exhibited a significant increase after 2 h (C1) of cold stress, compared with the control (Figure 8D,E). Furthermore, the KEGG pathway “arginine and proline metabolism” and “glutathione metabolism” were significantly enriched, according to the KEGG pathway enrichment analysis (Figure 7). Additionally, the expression profile of the related-proline and glutathione DEGs were analyzed, and most of these DEGs were enhanced in response to cold stress (Figures S9 and S10). These results suggested a high biological importance of the related-proline and glutathione DEGs in response to cold stress in alfalfa.

### 2.8. Transcriptional Regulatory Networks are Involved in the Adaptation of Alfalfa to Cold Stress

Transcription factors play important roles in the response to abiotic stresses and directly control the expression of specific sets of stress-responsive genes [45]. In this study, a total of 93 DEGs, which belonged to 27 gene families, were identified as transcription factors (TFs). Based on our data, the number of stress-induced TFs was much larger than the number of stress-suppressed TFs in all four treatments (Table S5). Additionally, only two and one stress-suppressed TFs appeared in the C1 and C2 groups, respectively. Among these TFs, the most abundant transcription factor family was *MYB* (13), followed by the *AP2-EREBP* (12), *GRAS* (12), *NAC* (7), *WRKY* (5), *HSF* (5), and *bHLH* (5) families. These results are consistent with previous reports on alfalfa [6,40], but the number of TFs is lower than that previously reported (158 and 350 TFs, respectively), which may be because the strict standard of absolute log2-fold change ≥ 2 was used in our study. The AP2/EREBP, NAC, and WRKY families are important groups of TFs and are involved in the tolerance of plants to abiotic stresses [46]. Previously, Shu et al. performed a transcriptome sequencing analysis of alfalfa in response to cold (4 °C) and freezing (−8 °C) stresses, and 26 AP2-EREBP TFs were identified [40]. Most of these 26 AP2-EREBP TFs were up-regulated under cold and/or freezing stress, and similar results appear in our study (Figure S11). Moreover, most members of the WRKY families were up-regulated under cold treatments, and all NAC TFs were up-regulated during cold stress (Figure S11). In addition, different members of the MYB, HSF, and bHL families were either up- or down-regulated under cold treatments. The similar expression patterns of these families were reported in *Ammopiptanthus mongolicus*, which was sequenced using pooled mRNA extracted from drought-stressed, cold-stressed, and unstressed seedlings, as well as leaves from naturally grown shrubs [46]. In summary, sophisticated transcriptional regulation could participate in the adaptation of alfalfa to cold stress, and several families, particularly the AP2/EREBP, NAC, and WRKY families, may be important in these processes.

### 2.9. Plant Hormone-Related DEGs

Plant hormones are small molecular weight compounds and signaling molecules, which regulate a wide range of metabolic and development processes at extremely low concentrations [38]. Current evidence suggests that multiple mechanisms are involved in hormone-mediated abiotic stress responses in plants, and it is clear that hormones act as central regulators of cold stress responses in plants [6,21]. In the present study, the “plant hormone signal transduction” category was significantly enriched in the KEGG pathway enrichment analysis for all DEGs. This KEGG pathway category contains a total of 82 DEGs, which includes 10, 7, 5, 15, 5, and 6 ABA, auxin, brassinosteroids, ethylene, jasmonate, and phytochrome-related genes, respectively (Table S6).

Abscisic acid is an isoprenoid hormone and important stress hormone, which responds to cold and freezing stress in plants. Current evidence suggests that the exogenous application of abscisic acid promotes freezing tolerance. Furthermore, abscisic acid mutants exhibited an altered cold resistance [21]. Protein phosphatase 2C (PP2C) is a regulator of the ABA signal transduction pathway and negatively regulates ABA responses and mitogen-activated protein kinase (MAPK) cascade pathways, which play important roles in stress signal transduction in plants [47]. Hu et al. over-expressed the *ZmPP2C2* gene in tobacco under the control of the Cauliflower Mosaic Virus (CaMV) 35S promoter and assessed various physiological changes in wild-type and transgenic plants under low temperatures [48]. The results showed that the over-expression of *ZmPP2C2* in tobacco enhanced its tolerance to cold stress. In addition, the expression pattern of *PP2C* genes was up-regulated under cold or freezing stress in alfalfa and *Populus euphratica* [6,8]. In this study, a total of three *PP2C* genes ([Msa]_25235, [Msa]_38071, and [Msa]_44978) were found in the “plant hormone signal transduction” category (Table S6), and the expression pattern of these three genes was up-regulated during 48 h of cold stress treatment (Figure S12). These results again validated that the stimulation of the ABA signaling pathway in alfalfa under cold stress is similar to the response of *P. euphratica* to freezing stress and that *PP2C* may be involved in ABA-dependent pathways.

In addition to ABA, other hormone-related biological processes were significantly enriched among the DEGs under cold stress, such as brassinosteroids (BRs), jasmomc acid (JA), and ethylene. BRs are polyhydroxylated steroids and closely interact with GAs, which regulate plant growth and development through a protein complex that includes the leucine-rich repeat receptor-like protein kinase (LRR-RLK) and brassinosteroid-insensitive 1 (BRI1) [49]. Researchers showed that a novel plant-specific LRR-RLK from *Glycine soja* (*GsLRPK*) was cold-inducible and that the overexpression of *GsLRPK* in yeast and *Arabidopsis* can enhance its resistance to cold stress and increase the expression of a number of cold responsive gene markers [50]. We found that the expression of LRR-RLK-related DEGs ([Msa]_27204, [Msa]_28587, and [Msa]_50481) was increased in this study (Figure S12 and Table S6). Thus, these genes may enhance the cold resistance of alfalfa.

### 2.10. Cold Stress-Related Genes Functionally Classified through MapMan Analysis

To classify these DEGs, we primarily used the metabolism overview, photosynthesis, and chloroplast custom array, installed in the MapMan tool kit, which allows one to group genes into different functional categories and visualize data through diagrams [51]. In the metabolism overview, starch and sucrose metabolism (100 genes), flavonoids metabolism (97), and light reactions (88) were the most common (Figure S13). The predominance of the starch and sucrose metabolism terms was well-matched with our finding that GO terms related to those processes were enriched in the biological process category (Figure 6), and it was also well-matched with the results of the KEGG enrichment analysis (Figure 7). This is consistent with a result in previous reports on alfalfa under cold stress [6]. In addition, our photosynthesis and chloroplast custom array showed that genes associated with PSI and PSII were most frequently coupled with cold stress-related genes, and a large number of these DEGs were down-regulated (Figure S14). Moreover, a decrease in chlorophyll content was observed in four groups (C1, C2, C3, and C4) under cold stress, compared with the control (Figure 1B). These results fully demonstrate that the photosynthesis of alfalfa is inhibited by cold stress, which is consistent with previous reports on paper mulberry and sorghum [52,53]. Photoinhibition might be the main adaptive mechanism of alfalfa in response to cold stress, and enhancing the transport and hydrolysis of photosynthetic products could be the potential target for improving the cold tolerance of alfalfa [53].

## 3. Materials and Methods

### 3.1. Plant Materials and Stress Treatments

This study was performed at Lanzhou University, Lanzhou, China. A hydroponic experiment was conducted to obtain the experimental samples using alfalfa variety Zhongmu No. 1, which is widely cultivated in the northern region. This variety was provided by the Qingchuan Yang Laboratory of the Beijing Institute of Animal Sciences, Chinese Academy of Agricultural Sciences, and it was cultivated by them. The vernalization of seeds was performed at 4 °C before germination, and the seedlings were transferred into a nutrient solution (1/2 MS, pH = 5.8) after three days of seed germination and grown under a 16 h light/8 h dark cycle at 22 °C. When the third leaf of alfalfa appeared (approximately ten days after germination), the seedlings were placed in an artificial climate incubator and frozen under a 16-h-light/8-h-dark cycle at 4 °C. There were five treatments performed on the alfalfa seedlings in this experiment, which included four cold treatment time points (2, 6, 24, and 48 h, which were represented by C1, C2, C3, and C4, respectively) and one control (0 h, which was represented by C0). To harvest the treated seedlings across all treatments at the same time, the cold stress for the different treatments was staggered. The initial cold stress was performed on the 48-h treatment and the control, and this was followed by the 24, 6, and 2 h treatments 24, 42, and 46 h later, respectively.

### 3.2. Physiological Experiments and RNA Extraction

For physiological experiments, a total of 60 seedlings (each replicate containing 20 seedlings) were harvested and pooled for each treatment group at the corresponding time point. Leaf materials were quickly harvested from the seedlings, and a subset of each sample was packaged to collect the chlorophyll content and electrolyte leakage data, as described previously [54,55]. The intact seedling, which includes the leaf and root, was flash-frozen in liquid nitrogen and stored at −80 °C. These materials were used for estimating other physiological indexes, such as the contents of MDA, H_2_O_2_, GSH, and Pro, and the enzyme activities of CAT and POD. The above-mentioned physiological indicators were ascertained according to the manufacturer’s instructions (CAT-2-Y, MDA-2-Y, GSH-2-W, H_2_O_2_-2-Y, POD-2-Y, PRO-2-Y; Komin, Suzhou, China; http://www.cominbio.com/). Each experiment was repeated at least three times. All data were subjected to a one-way analysis of variance (ANOVA), according to the model of a completely randomized design, using SPSS software (19.0, Chicago, IL, USA). The differences among the treatment means were evaluated by the Duncan’s multiple range test, with a 0.05 level of significance.

A total of six seedlings (each replicate containing two seedlings) were collected and pooled into a frozen tube pipe, for each treatment at the corresponding time point, and these were used for RNA extraction. The total RNA of each treatment was isolated using the Trizol method (Sangon Biotech, Shanghai, China), according to the manufacturer’s instructions. The concentration of each sample, determined using a NanoDrop ND1000 spectrophotometer (Thermo Scientific, Waltham, MA, USA), was greater than 600 ng/μL, for those samples submitted for transcriptome sequencing.

### 3.3. De Novo Assembly of Transcriptome

For RNA-seq, the intact alfalfa seedlings for each treatment were collected and pooled separately to prepare 15 cDNA libraries (three biological replicates for each treatment). The cDNA library was constructed using an mRNA-Seq Sample Preparation Kit, according to the manufacturer’s instructions (MGIEasy™ mRNA library preparation kit, MGI, Shenzhen, China). Poly(A) mRNA was enriched with oligo magnetic adsorption. We also fragmented the enriched mRNA and reversed the transcribed to a double-strand cDNA (dscDNA) with an N6 random primer. Sequencing adaptors were linked to the purified cDNA, and 15 double-strand libraries were obtained by PCR amplification. These libraries were sequenced on a BGISEQ-500 platform, made by the Beijing Genomic Institution (www.genomics.org.cn, BGI, Shenzhen, China).

All raw reads must be filtered before downstream analysis to decrease data noise because these reads may contain the adaptor sequence, a high content of unknown bases, and low-quality reads. Filtering included three steps: Remove reads with adaptors, remove reads in which unknown bases are more than 10%, and remove low quality reads. We used Bowtie2 to map the clean reads to the reference genes and used BWA for the reference genomes [56,57], which were obtained from a previous study of transcriptome sequencing, performed by PacBio ISO Sequencing.

### 3.4. Quantitative Real-Time PCR Analysis

The total RNA from all samples used for the RNA-Seq analysis was also used for qRT-PCR validation. The single-strand cDNAs, used for qRT-PCR, were synthesized from 2.5 µg of the total RNA, with MMLV reverse transcriptase (TaKaRa, Dalian, China). To verify the RNA-Seq results, nine unigenes were selected for the qRT-PCR test. QRT-PCR was performed using a SYBR Premix Ex Taq II Kit (TaKaRa, Dalian, China) on a 7500 Fast Real-time PCR system (Applied Biosystems, Foster City, CA, USA) under the following parameters: 95 °C for 30 s and 40 cycles of 95 °C for 5 s and 60 °C for 30 s. Gene-specific primers were designed using Primer Premier 6 software (Premier Biosoft International, Palo Alto, CA, USA) and are shown in Table S3. Each treatment time point included three samples. Three technical replicates were performed for each sample, and the relative expression levels were normalized to the expression of the *Medicago* actin gene (AA660796) and calculated using the 2^−ΔΔ*C*t^ method.

### 3.5. Differential Gene Expression, GO, and KEGG Enrichment Analysis

The FPKM method was used to calculate the unigene expression level [58]. DEGs between different treatments were identified with the NOISeq package [59]. DEGs were restricted, with the absolute value of fold change ≥ 4 and divergence probability ≥ 0.8 as the thresholds, by performing pairwise comparisons of cold treated samples with the control sample. To annotate the biological functions of the DEGs, the GO and KEGG pathway enrichment analyses for DEGs were performed using the GO database (http://geneontology.org/) and KEGG database (http://www.genome.jp/kegg/). In addition, the expression data υ (C0, C1, C2, C3, and C4) were normalized to 0, log2 (υC1/υC0), log2 (υC2/υC0), log2 (υC3/υC0), and log2 (υC4/υC0), and clustered using STEM software, with a *p* ≤ 0.05, to examine the expression profile of DEGs [60].

The cluster 3.0 program was used to draw a heatmap of the significant DEGs in response to short-term cold stress, and Java TreeView was used to view the clustering results generated by Cluster 3.0 (http://bonsai.hgc.jp/~mdehoon/software/cluster/software.htm). Moreover, the metabolic pathways of alfalfa in response to cold stress were plotted using MapMan, according to the default parameters (http://mapman.gabipd.org/).

## 4. Conclusions

To investigate the physiological and biochemical changes of alfalfa in response to cold stress, a total of eight related physiological indicators were measured, including electrolyte leakage, chlorophyll content, and six antioxidant indicators. As a result, the activity of CAT and POD, as well as the content of proline and GSH, increased by varying degrees. In the present study, using cold stress-treated alfalfa samples at five time points, we performed a gene expression comparison at the transcriptional scale using RNA-Seq. In total, 50,809 unigenes were obtained from 15 libraries (three libraries for each time point), and 5283 DEGs were identified from cold stress treated alfalfa samples. Then, these DEGs were analyzed with regard to their potential role in the response to cold stress using clustering, GO, and KEGG enrichment analyses. Most of the genes were involved in carbohydrate metabolism, photosynthesis, plant hormone signal transduction, and the biosynthesis of amino acids and were significantly enriched. Furthermore, the antioxidant defense system plays important roles in the alfalfa response to cold stress, as demonstrated by the expression pattern of the related genes during a 48-h cold stress treatment. As a result, a detailed investigation of the pathways and candidate genes was provided, which enhances the understanding of the molecular mechanisms underlying alfalfa responses to cold stress and fall dormancy and offers a wide range of genetic resources for breeders.

## Supplementary Materials

Supplementary materials can be found at http://www.mdpi.com/1422-0067/19/10/3169/s1.

## Abbreviations

FPKM	Fragments Per Kilobase Of Exon Per Million Fragments Mapped
Nr	NCBI non-redundant protein sequences
GO	Gene Ontology
KEGG	Kyoto Encyclopedia of Genes and Genomes
qRT-PCR	Quantitative real-time PCR
GSH	Glutathione
Pro	Proline
CAT	Catalase
POD	Peroxidase
NGS	Next-generation sequencing
DEGs	Differentially expressed genes
ROS	Reactive oxygen species
H_2_O_2_	Hydrogen peroxide
MDA	Malondialdehyde
TFs	Transcription factors
ABA	Abscisic acid
STEM	Short Timeseries Expression Miner
ANOVA	One-way analysis of variance
