# Supplementary figures and images for: Multiple Regulatory Networks Are Activated during Cold Stress in Medicago sativa L
Source: Int J Mol Sci. 2018 Oct 15;19(10):3169. doi: 10.3390/ijms19103169 (PMC6214131; doi:10.3390/ijms19103169)

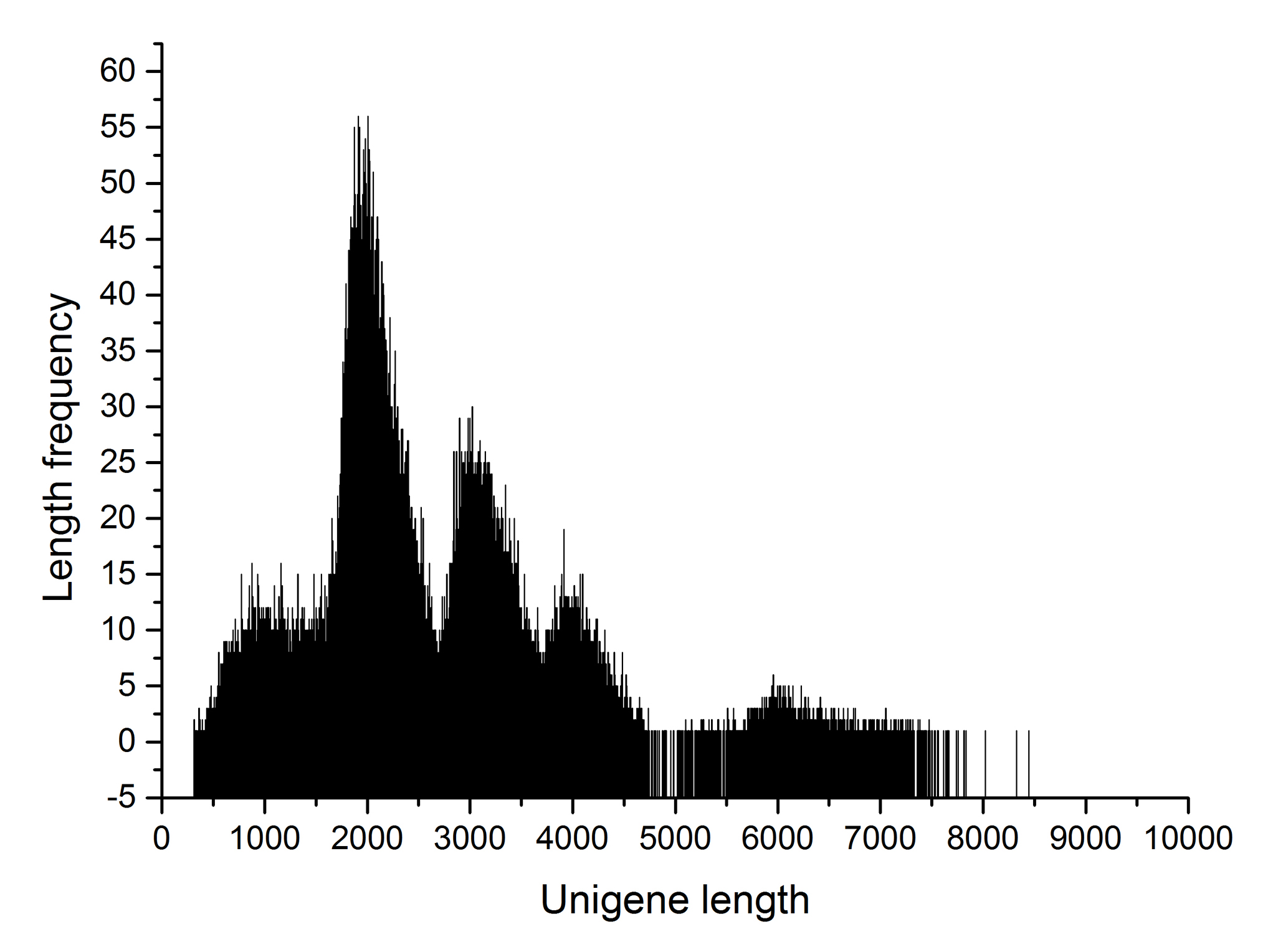

Supplement: Supplementary file 1 [file ijms-19-03169-s001.zip › supplementary material/Figure S1.jpg]

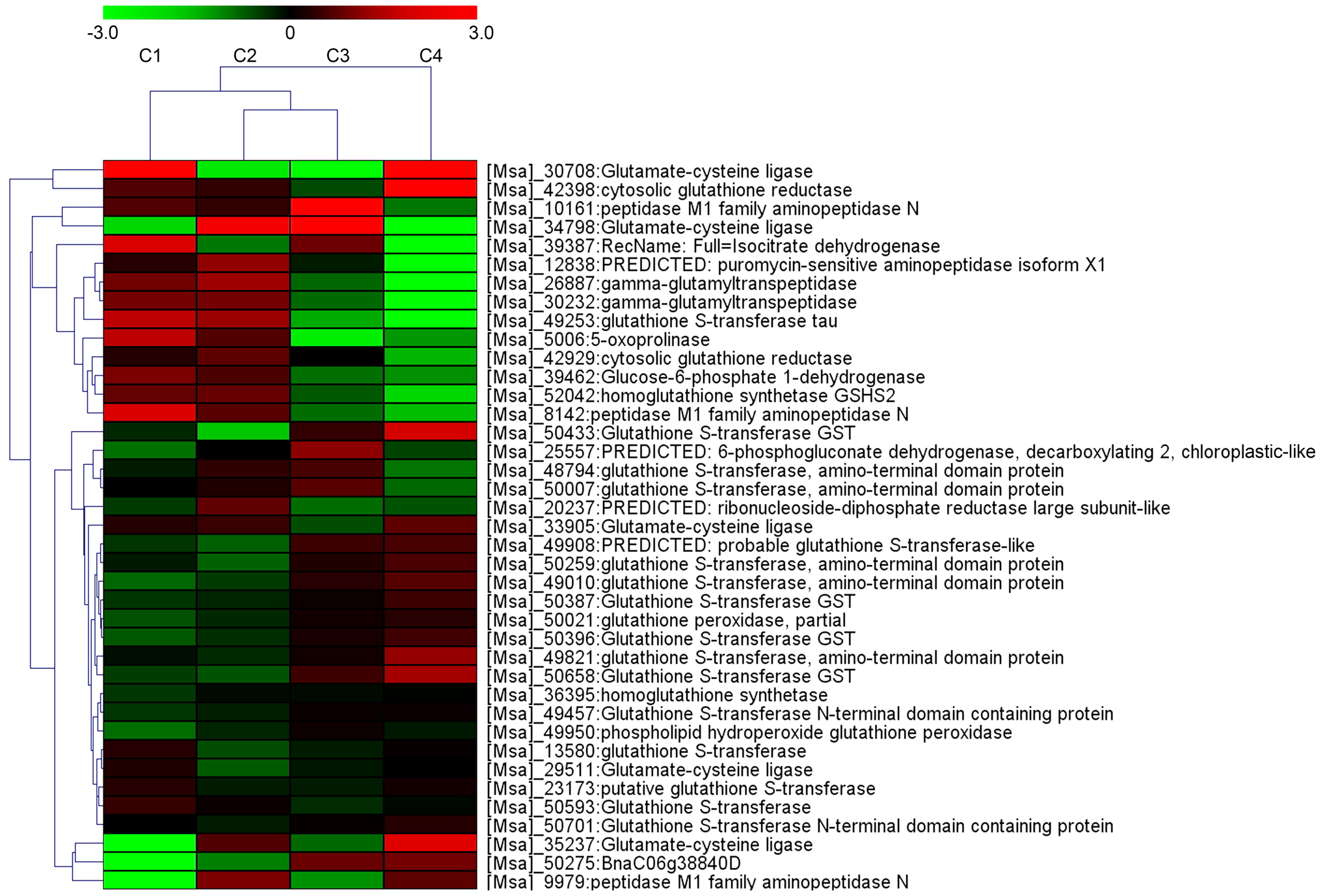

Supplement: Supplementary file 1 [file ijms-19-03169-s001.zip › supplementary material/Figure S10.jpg]

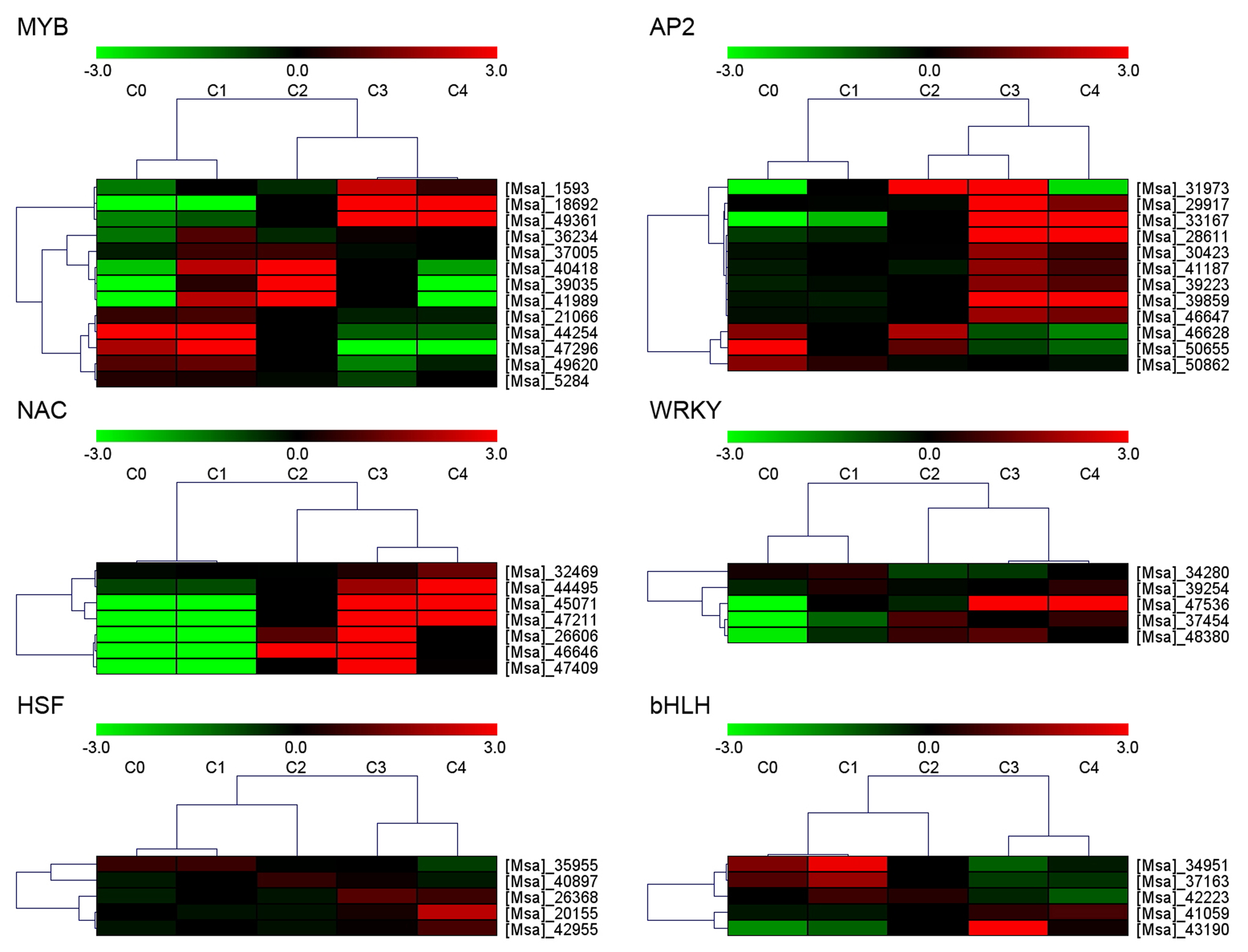

Supplement: Supplementary file 1 [file ijms-19-03169-s001.zip › supplementary material/Figure S11.jpg]

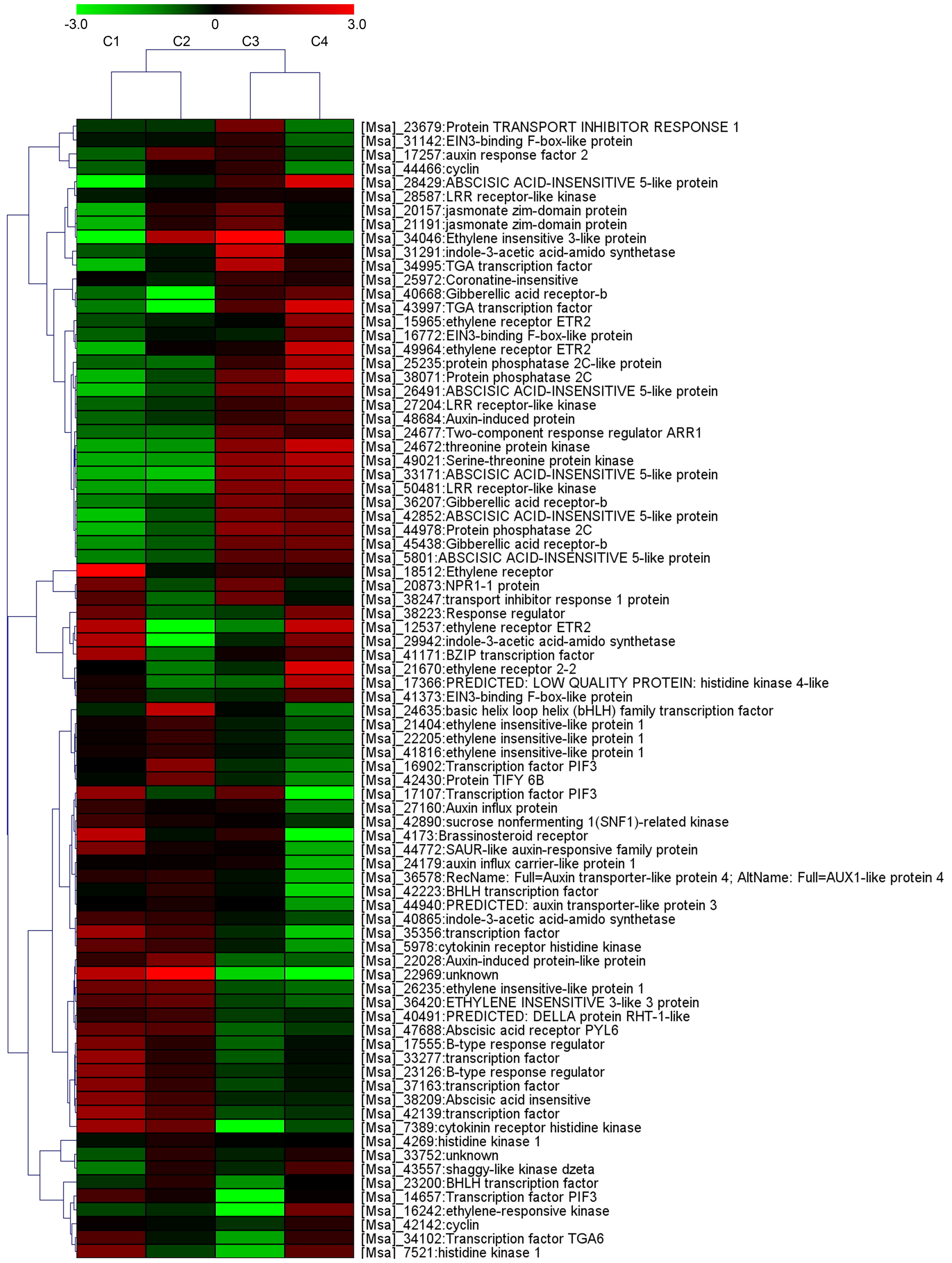

Supplement: Supplementary file 1 [file ijms-19-03169-s001.zip › supplementary material/Figure S12.jpg]

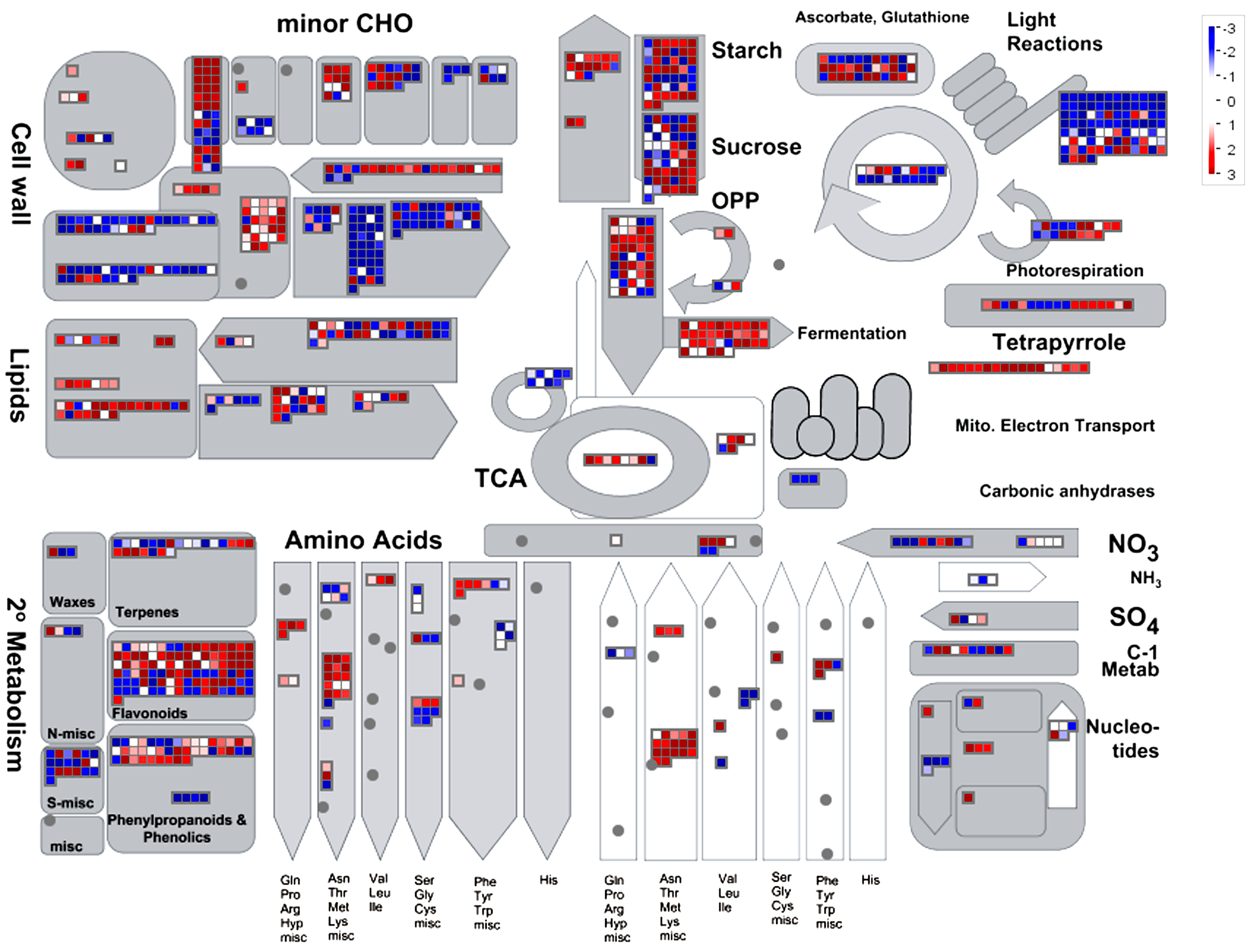

Supplement: Supplementary file 1 [file ijms-19-03169-s001.zip › supplementary material/Figure S13.jpg]

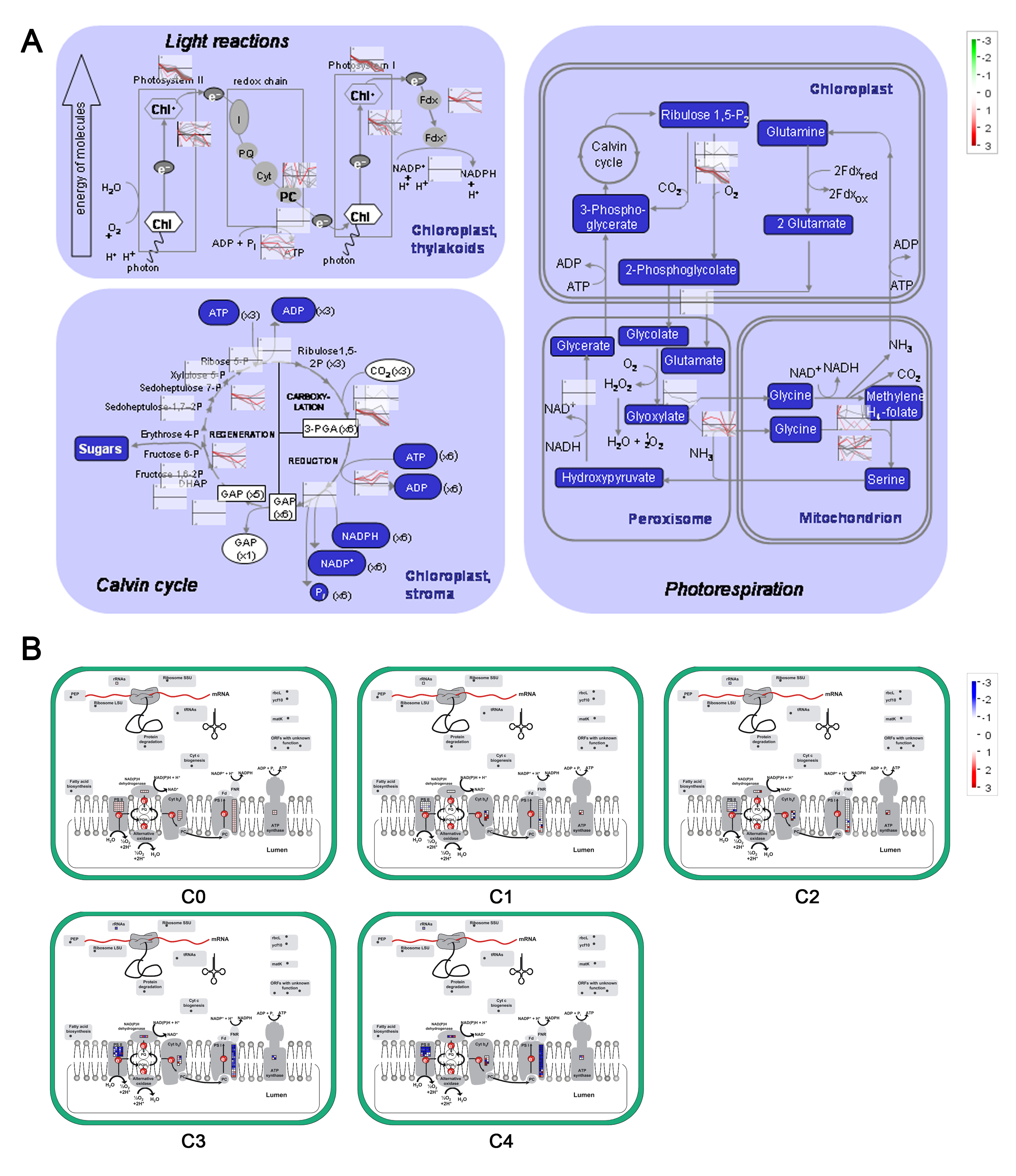

Supplement: Supplementary file 1 [file ijms-19-03169-s001.zip › supplementary material/Figure S14.jpg]

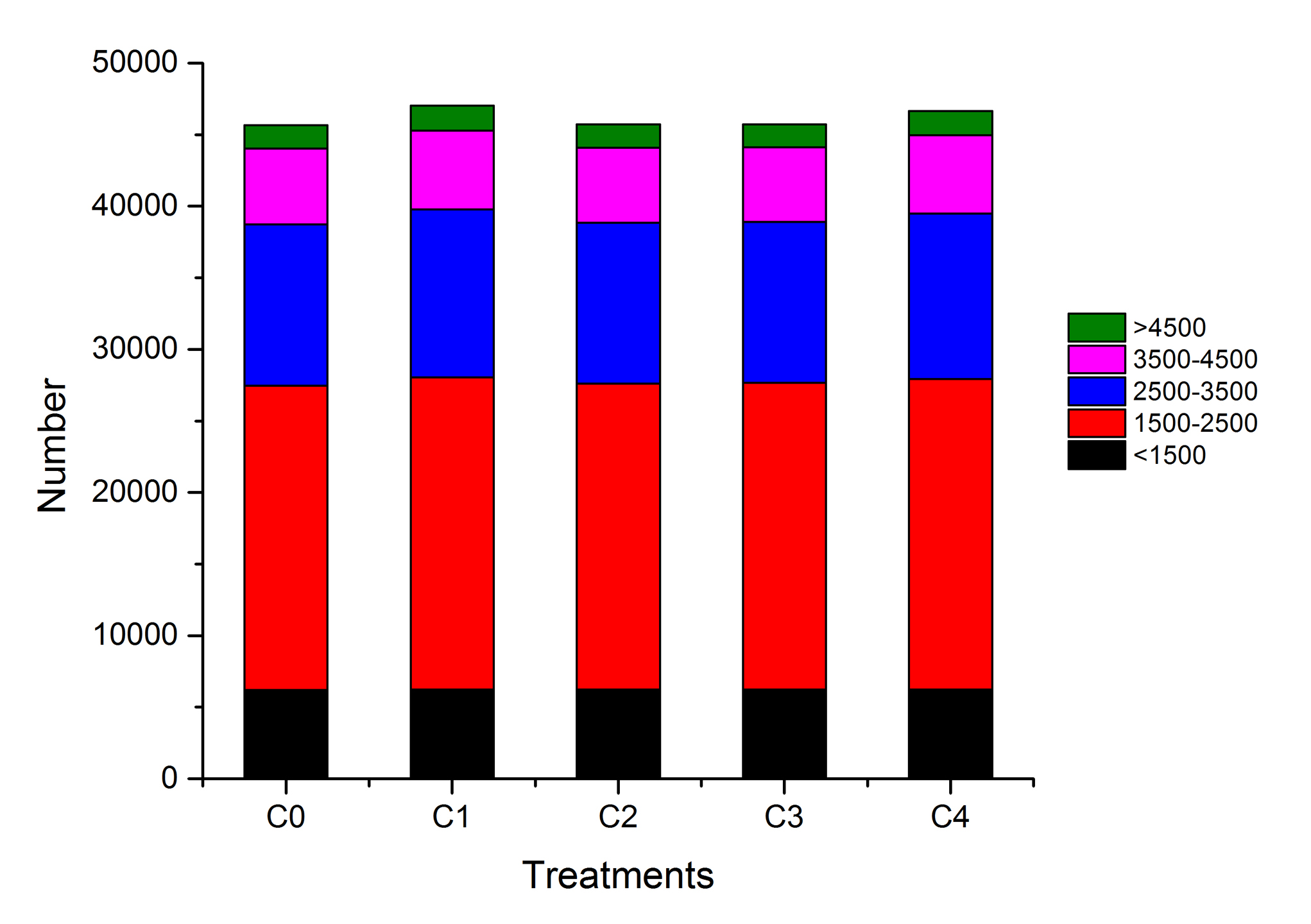

Supplement: Supplementary file 1 [file ijms-19-03169-s001.zip › supplementary material/Figure S2.jpg]

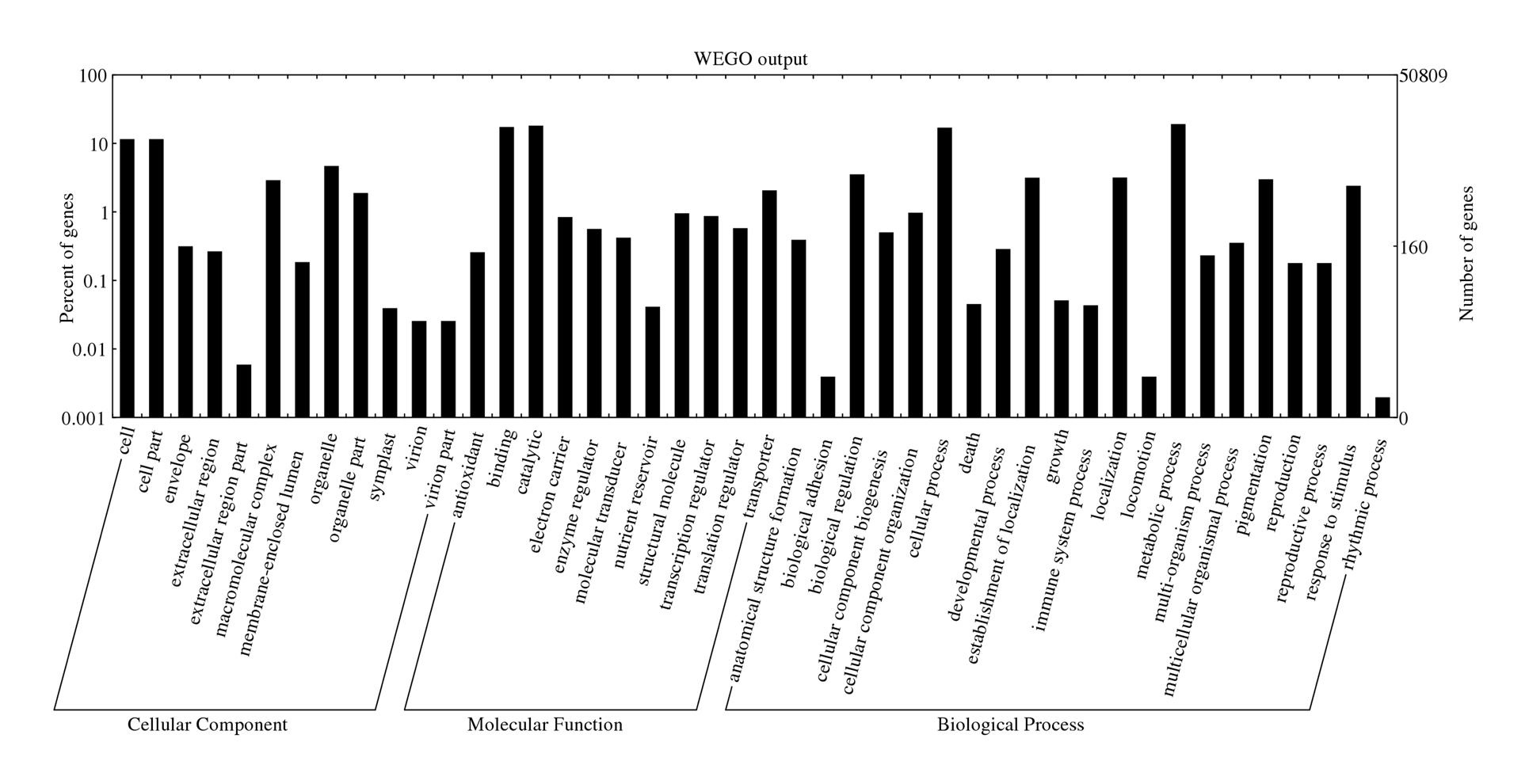

Supplement: Supplementary file 1 [file ijms-19-03169-s001.zip › supplementary material/Figure S3.jpg]

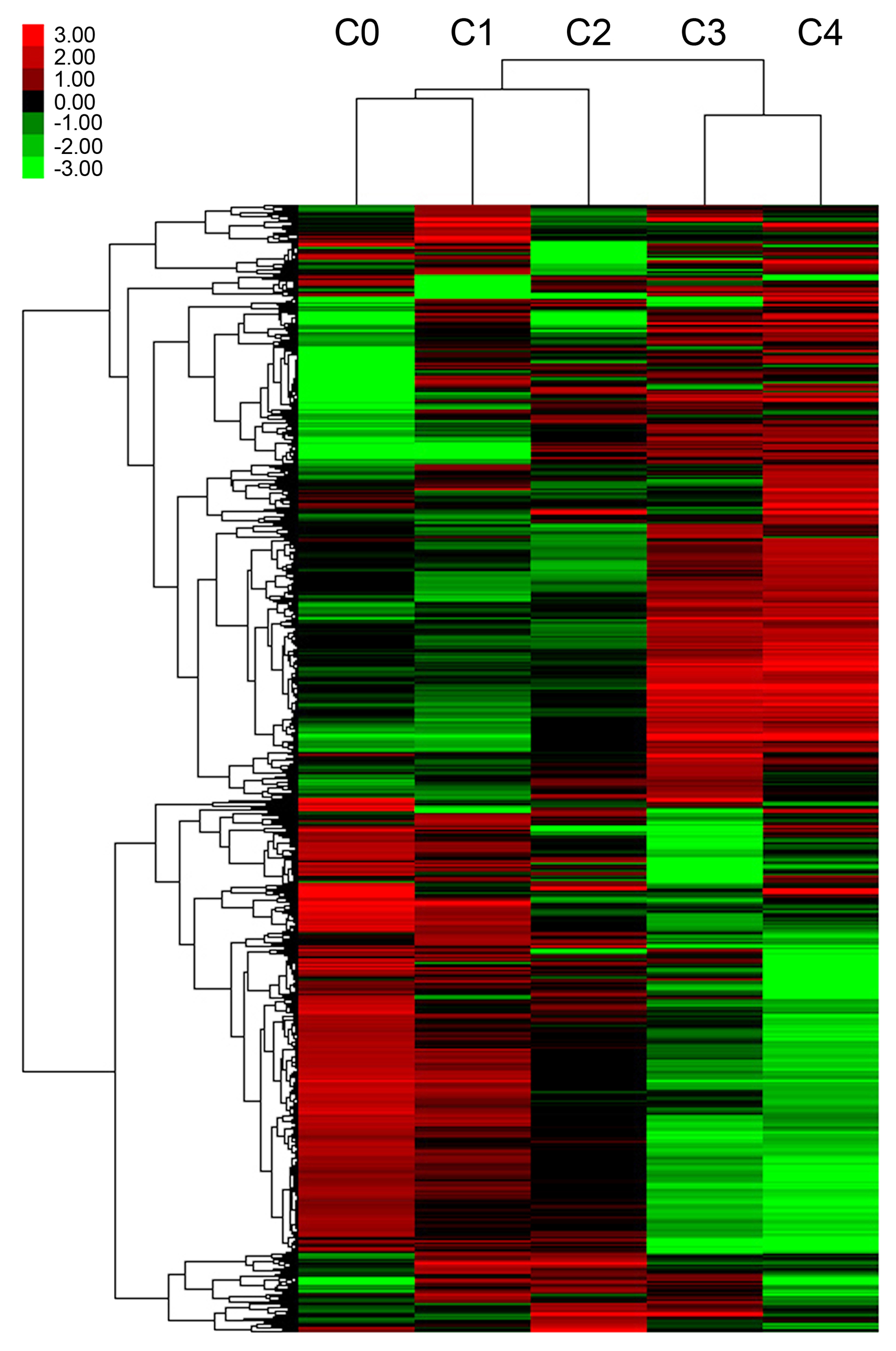

Supplement: Supplementary file 1 [file ijms-19-03169-s001.zip › supplementary material/Figure S4.jpg]

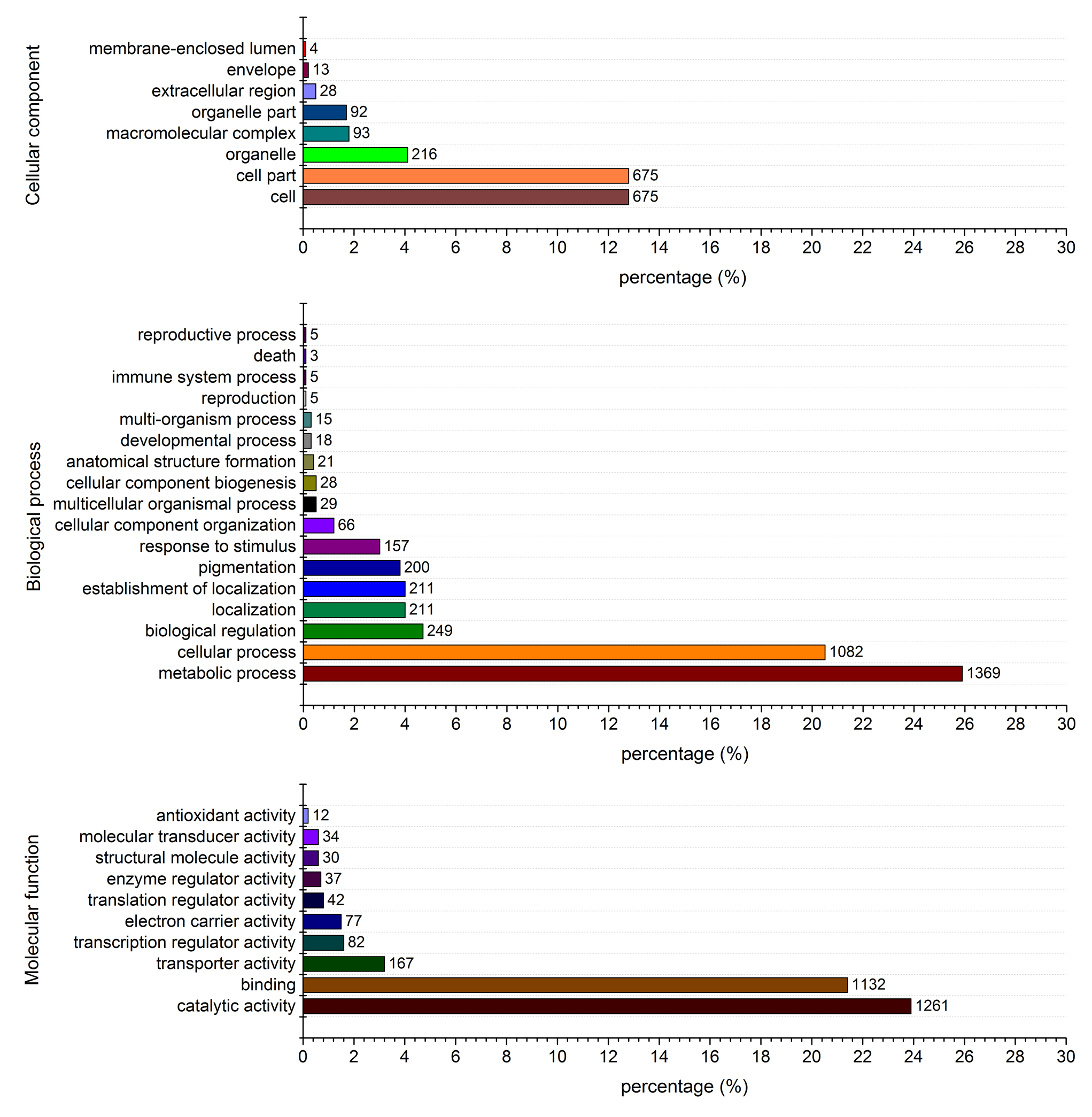

Supplement: Supplementary file 1 [file ijms-19-03169-s001.zip › supplementary material/Figure S5.jpg]

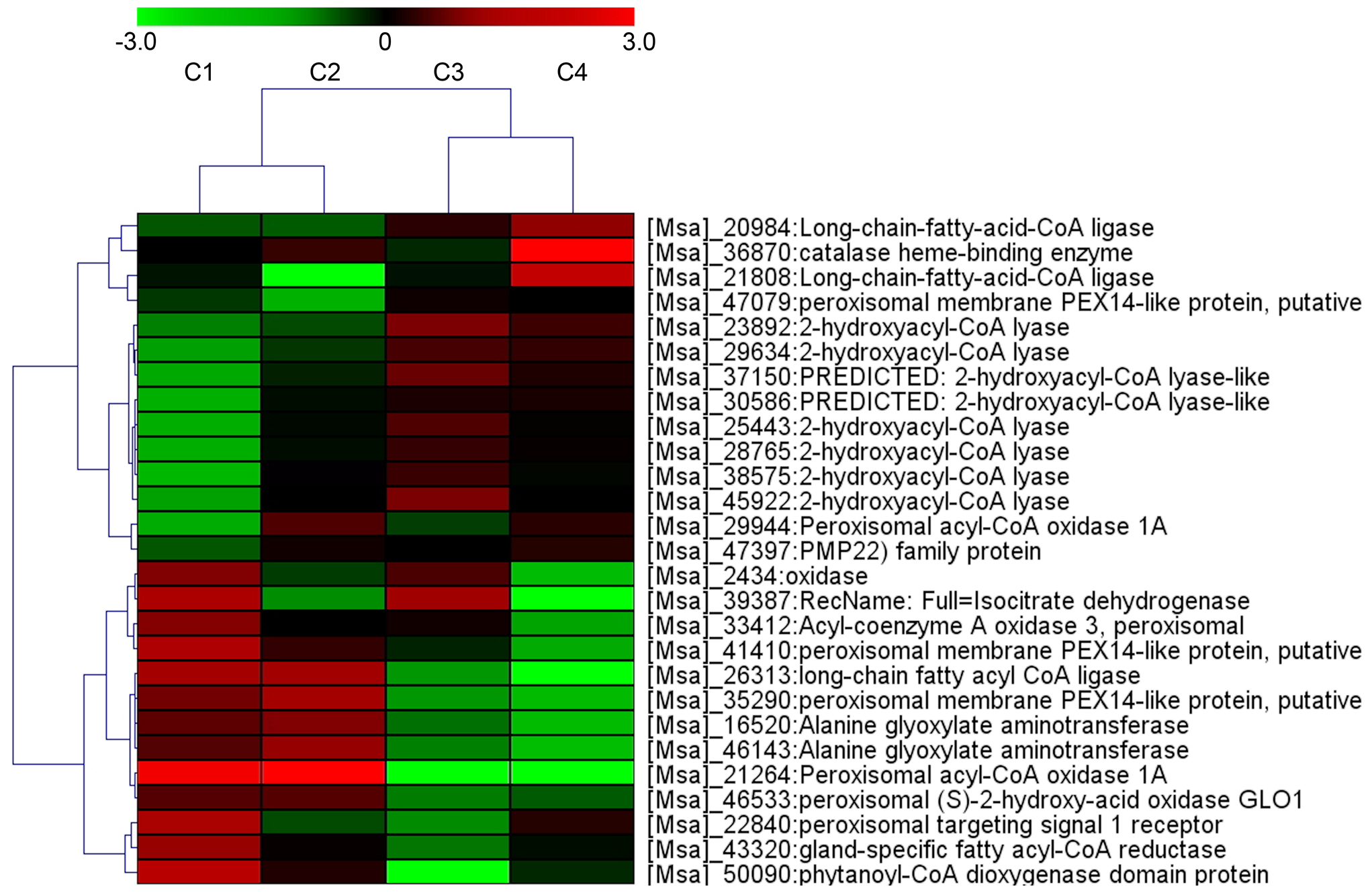

Supplement: Supplementary file 1 [file ijms-19-03169-s001.zip › supplementary material/Figure S6.jpg]

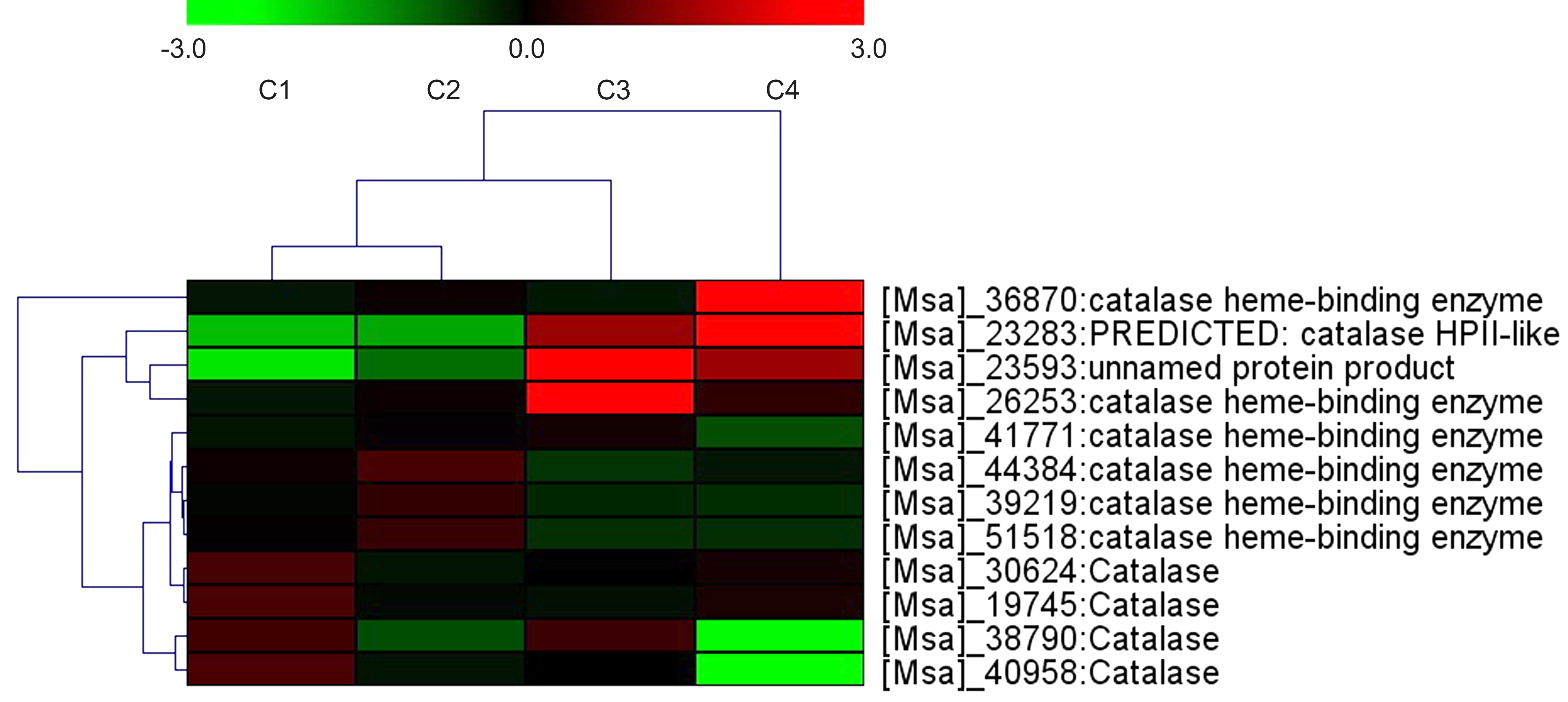

Supplement: Supplementary file 1 [file ijms-19-03169-s001.zip › supplementary material/Figure S7.jpg]

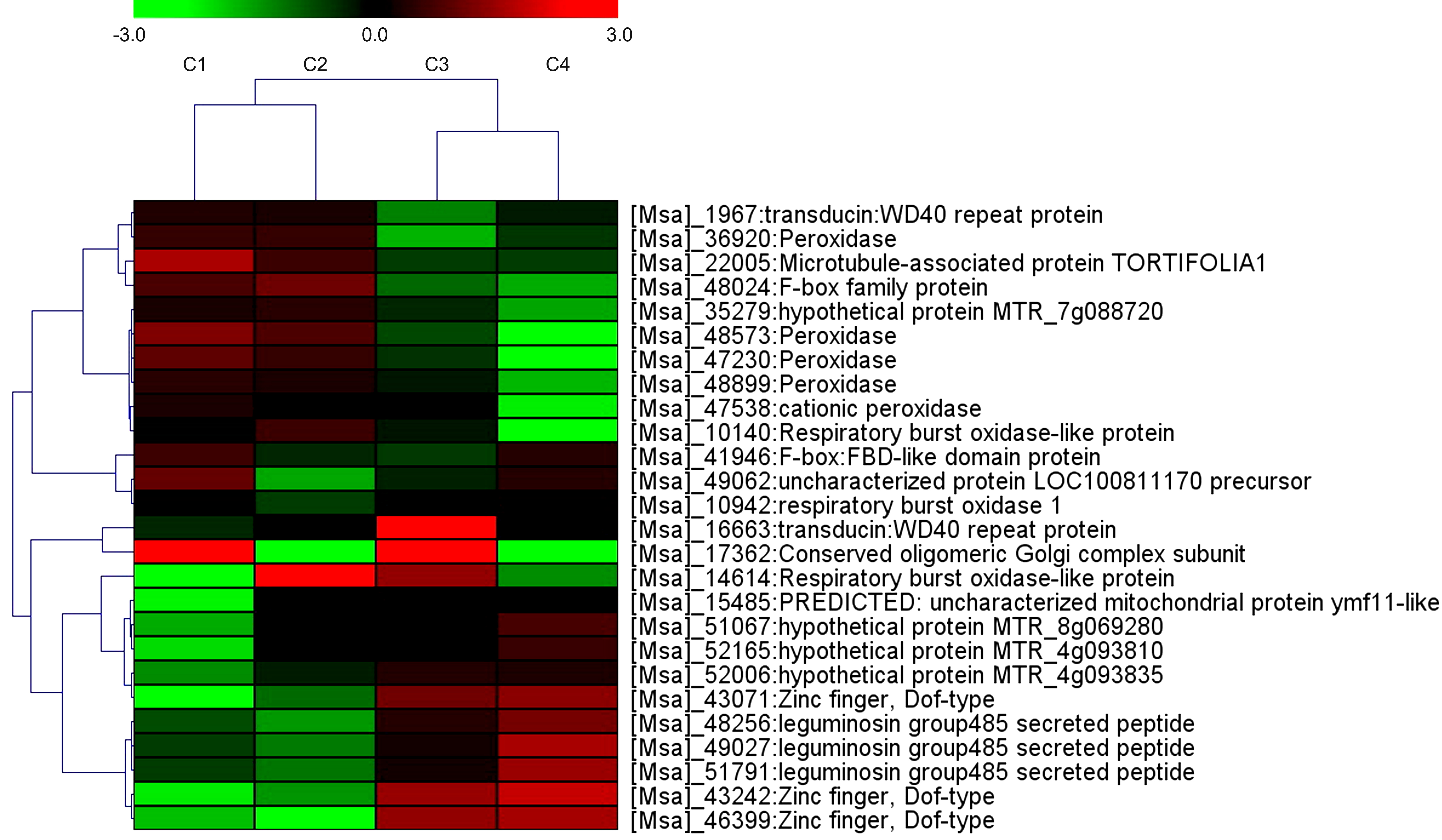

Supplement: Supplementary file 1 [file ijms-19-03169-s001.zip › supplementary material/Figure S8.jpg]

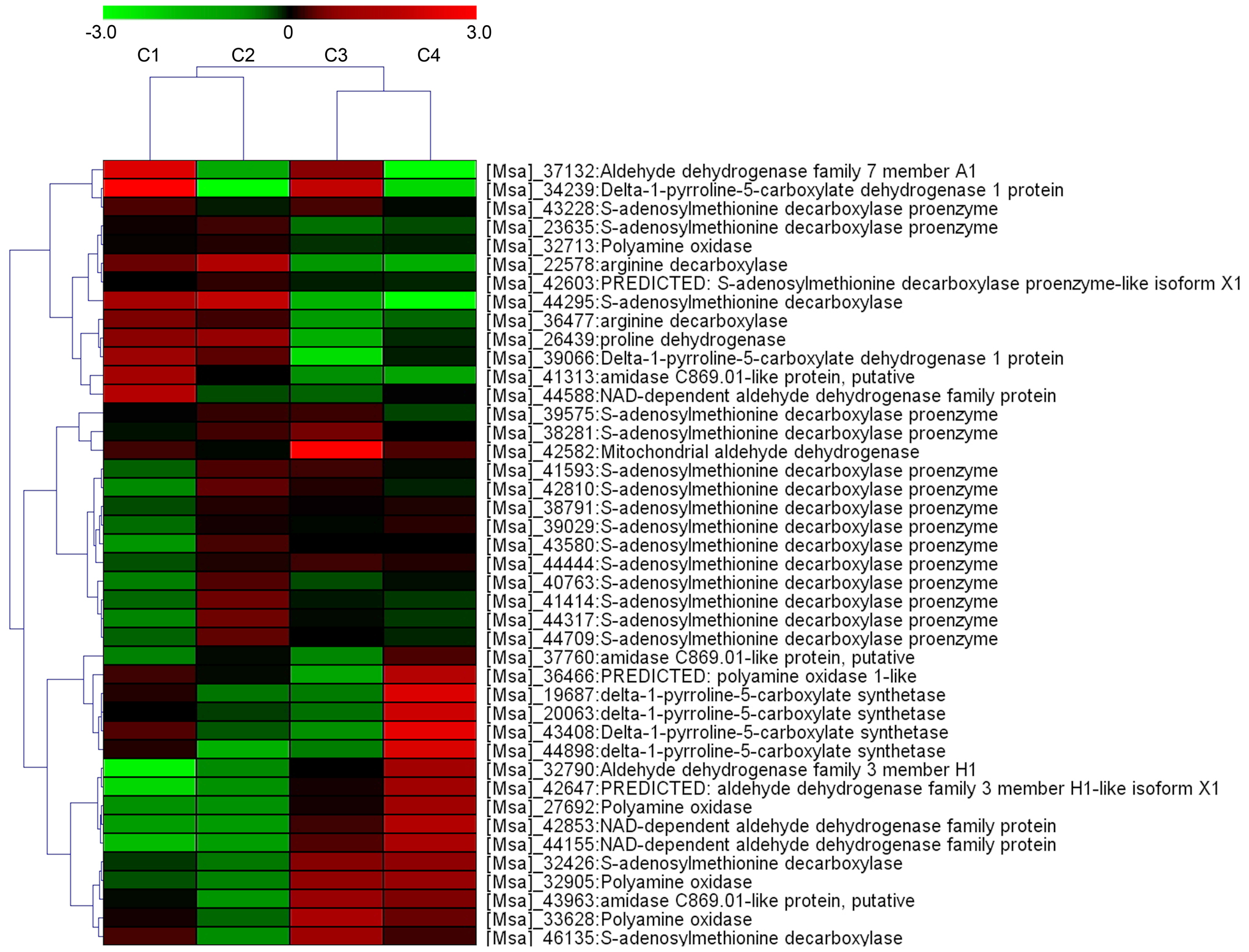

Supplement: Supplementary file 1 [file ijms-19-03169-s001.zip › supplementary material/Figure S9.jpg]
